# Devices for Self-Monitoring Sedentary Time or Physical Activity: A Scoping Review

**DOI:** 10.2196/jmir.5373

**Published:** 2016-05-04

**Authors:** James P Sanders, Adam Loveday, Natalie Pearson, Charlotte Edwardson, Thomas Yates, Stuart JH Biddle, Dale W Esliger

**Affiliations:** ^1^ Leicester-Loughborough Diet, Lifestyle and Physical Activity Biomedical Research Unit Loughborough University Loughborough United Kingdom; ^2^ National Centre for Sport and Exercise Medicine School of Sport, Exercise and Health Sciences Loughborough University Loughborough United Kingdom; ^3^ Diabetes Research Centre College of Medicine, Biological Sciences and Psychology University of Leicester Leicester United Kingdom; ^4^ Active Living and Public Health Institute of Sport, Exercise & Active Living (ISEAL) Victoria University Melbourne Australia

**Keywords:** sitting time, physical activity, measurement, feedback, activity monitor, scoping review

## Abstract

**Background:**

It is well documented that meeting the guideline levels (150 minutes per week) of moderate-to-vigorous physical activity (PA) is protective against chronic disease. Conversely, emerging evidence indicates the deleterious effects of prolonged sitting. Therefore, there is a need to change both behaviors. Self-monitoring of behavior is one of the most robust behavior-change techniques available. The growing number of technologies in the consumer electronics sector provides a unique opportunity for individuals to self-monitor their behavior.

**Objective:**

The aim of this study is to review the characteristics and measurement properties of currently available self-monitoring devices for sedentary time and/or PA.

**Methods:**

To identify technologies, four scientific databases were systematically searched using key terms related to behavior, measurement, and population. Articles published through October 2015 were identified. To identify technologies from the consumer electronic sector, systematic searches of three Internet search engines were also performed through to October 1, 2015.

**Results:**

The initial database searches identified 46 devices and the Internet search engines identified 100 devices yielding a total of 146 technologies. Of these, 64 were further removed because they were currently unavailable for purchase or there was no evidence that they were designed for, had been used in, or could readily be modified for self-monitoring purposes. The remaining 82 technologies were included in this review (73 devices self-monitored PA, 9 devices self-monitored sedentary time). Of the 82 devices included, this review identified no published articles in which these devices were used for the purpose of self-monitoring PA and/or sedentary behavior; however, a number of technologies were found via Internet searches that matched the criteria for self-monitoring and provided immediate feedback on PA (ActiGraph Link, Microsoft Band, and Garmin Vivofit) and sedentary time (activPAL VT, the Lumo Back, and Darma).

**Conclusions:**

There are a large number of devices that self-monitor PA; however, there is a greater need for the development of tools to self-monitor sedentary time. The novelty of these devices means they have yet to be used in behavior change interventions, although the growing field of wearable technology may facilitate this to change.

## Introduction

Modern environments and technological advancements have radically altered the way we live our lives [1]. The need to undertake purposeful physical activity (PA) has all but disappeared and sedentary behavior, defined as “any waking behavior in a sitting or reclining posture with an energy expenditure ≤1.5 metabolic equivalent” [2] is the dominant behavior. Low levels of moderate-to-vigorous PA (MVPA) have been consistently associated with the risk of developing chronic diseases, such as type 2 diabetes, cardiovascular disease, and some cancers [3]. In addition, increasing the total level of daily movement, such as the number of steps taken, has been strongly inversely associated with the risk of developing chronic diseases [4,5]. There is also mounting evidence that the amount of time spent sedentary is an important determinant of health status independent of PA levels. For example, Wilmot and colleagues [6] found that when comparing those with the highest levels of sedentary behavior to those with the lowest levels, independent of PA levels, there was a 112%, 147%, 90%, and 49% increase in the relative risk of type 2 diabetes, cardiovascular disease, cardiovascular mortality, and all-cause mortality, respectively. Moreover, how sedentary time and PA are accumulated throughout the day may also be important, with frequent breaks in sedentary behavior associated with a healthier metabolic profile [7]. This has necessitated a paradigm shift that focuses on both the accumulation of MVPA (the traditional focus of lifestyle interventions) and the importance of postural allocation throughout the waking hours.

Over the last decade, there has been a plethora of tools developed to support PA and sedentary behavior change, of which the greatest growth has been seen in self-monitoring tools. Self-monitoring is defined as “a person closely and deliberately monitors their own behavior” [8,9] and “allowing the modification of their behaviors to achieve predetermined goals or outcomes” [10] and has a strong theoretical foundation for behavior change. Self-regulation theory posits that self-monitoring precedes self-evaluation of progress made toward one’s goal and as well as preceding self-reinforcement of behavior for progress to be made [9]. Furthermore, Control Theory proposes that self-monitoring of behavior, setting goals, receiving feedback, and reviewing relevant goals with feedback work synergistically and are central to self-management and behavioral control [11,12]. Self-monitoring, therefore, can increase an individual’s personal responsibility, promote independence, and individuals can create their own pathways toward goal achievement by taking an active rather than passive role [13]. When included in behavior change interventions, self-monitoring has proven to be an effective behavior change strategy across a variety of behaviors, including smoking, diet, and PA, and it is considered a foundation of lifestyle behavior change interventions [12,14].

Traditionally, self-monitoring of PA and sedentary time occurred via paper-based journal methods [14]; more recently, the pedometer became a popular method of self-monitoring for interventions designed to increase PA. Individuals who used pedometers increased their PA by 26.9% from baseline activity levels [15]. Subsequently, advances in technology have led to a proliferation in the number of bodily worn electronic devices becoming available that go beyond simply measuring and providing feedback on the number of steps per day (eg, Fitbit, Jawbone). Along with PA, electronic devices are also starting to measure sitting time, provide real-time feedback, as well as encouraging interruptions in prolonged sitting. It has been suggested that the use of these electronic approaches to self-monitor might lessen the burden of traditional methods and may improve adherence to self-monitoring resulting in greater achievement toward behavioral goals [16].

This increased availability of electronic self-monitoring devices provides an opportunity for researchers to utilize these novel technologies as an aid for behavior change in PA and sedentary behavior on a large scale. Furthermore, wearable technologies are increasingly integrating health care systems. Recent reports from the National Information Board in a review of the National Health Service in the United Kingdom indicate the need for “citizens” to start playing a more active role in their health care by accessing, entering, and uploading data into their own online medical record. Under these new plans, citizens will be able to access and download their detailed medical records as well as contribute to it with information from their personal wearable technology or biosensors [17,18]. In addition, as more health care providers in the United States move to a value-based care system (ie, “reward points” for positive lifestyle alterations that can be redeemed for discounts on a range of products and/or activities), mobile technologies that promote health and well-being by engaging in important health behaviors (eg, increased MVPA) will continue to grow and have the potential to be an integral piece of future health care systems. In light of this, a review of the current tools used to self-monitor PA and/or sedentary time has the potential to be a valuable resource to researchers, clinicians, health care providers, and the general public.

Therefore, it seems timely to review the characteristics and measurement properties (eg, wear location, integrated sensors, outcomes measured) of currently available self-monitoring devices, both those marketed to consumers and those used in research settings, that have been (or could be) utilized in, or developed for, real-time self-monitoring of sedentary behavior and/or PA.

## Methods

### Searches

The search strategy was built around three groups of keywords: behavior (ie, PA and sedentary behavior), measurement, and population. A detailed description of the keywords used and method of combination can be found in Multimedia Appendix 1. For the purposes of this study, tools were deemed to measure sedentary time if they could measure the wearer’s sitting and/or reclining posture.

Scopus, Medline, Web of Science, and the Institute of Electrical and Electronic Engineers (IEEE) databases were searched using these keywords from the inception of the databases to October 1, 2015. In addition, manual searches of personal files were conducted and reference lists of primary studies were screened.

### Internet Search Engines

Because of the rapid release of technology in the consumer electronic area, a grey literature search of relevant websites was conducted for technologies that allow for the self-monitoring of PA and sedentary time but may not have made it into the published research to date. Keywords based on the same groups as the database searches were used to search the Internet engines Google, Bing, and Yahoo. Searches were extracted for later review using a specialized browser plug-in. The first 200 search results from each search engine were extracted for further review; this was a pragmatic approach because it was deemed that results after the first 200 were either not relevant or repetitive. This ensured that the results were unaffected by the changing algorithms of Web search engines. Searches were completed on October 1, 2015.

### Study Inclusion and Exclusion Criteria

Two sets of inclusion criteria were developed for research articles and websites. For inclusion in the review, studies were required to (1) include adults aged 18 years or older, (2) be published in English, and (3) describe a device that objectively self-monitors PA, physical inactivity, and/or sedentary time/sitting and can, or has the potential to, provide feedback to the user. Traditionally, there would also be a criteria based around study type; however, in order to obtain the widest variety of devices, this was not included.

For inclusion in the review, only websites from manufacturers were included (ie, blogs or consumer reviews pertaining to technologies of interest were excluded) and devices that had the ability to self-monitor and were available for purchase at the time of the review were included.

### Data Extraction

Potentially relevant articles were selected by screening titles, screening abstracts, and if abstracts were not available or did not provide sufficient data, the entire article was sought and screened to determine whether it met the inclusion criteria. Relevant websites were selected by screening webpage titles and screening devices on relevant webpages to determine whether it met the inclusion criteria. Data were extracted on standardized forms developed for this review.

Information on the devices was extracted from articles and cross-referenced with the device manufacturer’s information. Validity data on each device was not extracted; instead, articles with relevant validity data, where available [19-38], were referenced in the data table because the authors chose to focus this review on the characteristics of the devices to allow the reader to make a judgment about their efficacy as self-monitoring tools.

A 10% subsample of potentially relevant articles retrieved for full-paper screening were extracted by a second author (AL) to determine interrater agreement. Interrater agreement was high (Cohen’s kappa=.81). If any discrepancies arose, these were resolved by discussion between authors.

### Self-Monitor Scoring

Each device was designated a self-monitoring code: (1) yes, self-monitors PA (Y_PA_); (2) yes, self-monitors PA and physical inactivity, such as self-monitoring and feedback on lack of movement (Y_PI_); and (3) yes, self-monitors sedentary time (Y_ST_).

The different attributes of the self-monitoring devices were based on Control Theory [11]; specifically, the ability to receive feedback (defined as the provision of informative and actionable insights on the performance of the behavior) and the ability to set goals (defined as agreeing on a goal/target defined in terms of the behavior to be achieved) [8]. Aspects included the different types of feedback (eg, vibratory, auditory, omnipresent in the form of colors or lights, or potentially via push notifications). Also included was the timing of the feedback (ie, immediate or delayed). Other features included the way in which the data were portrayed (eg, numeric data/graphical representation of the data). The platform pervasiveness was also included (ie, number of different devices/operating systems the data could be viewed on). Each of these was broken into the feedback attributes that were available on either the device or the backend platform (defined as the smart device/software that the technology connected to). Other attributes included were goal-setting capability of the device and whether the device or associated software could be customized by the end user via some method, usually an application programming interface or software development kit. Textbox 1 provides a detailed description of each self-monitoring attribute. Each attribute was split into whether the attribute was present on the device itself (denoted with “D”) or whether it was present on the backend platform (ie, mobile phone/tablet; denoted with “BP”).

**Description of the self-monitoring attributes coded.**

Auditory: feedback on behavior provided verbally from device (eg, via Sensoria voice-over feedback regarding ground contact from smartphone/smart MP3)

Vibratory: haptic feedback on predetermined behavioral thresholds provided using vibrations (eg, LumoBack)

Omnipresent: feedback that is visible all the time, usually in the form of a progression bar that changes with advancement toward predetermined goals (eg, Fitbit Flower)

Push notification: the delivery of information regarding behavioral goals from a software app to a computing device without a specific request from the user

Immediate: whether the data/feedback are immediate in its return to the user (eg, LumoBack)

Delayed: whether the data/feedback are delayed in its return to the user (eg, ActiGraph)

Numeric: data are returned in the form of numbers/figures or statistics

Graph: data are returned in the form of graphical representation

Written/textual feedback: data are returned in the form of textual feedback

‘Ometer (omnipresent meter): data are returned in the form of a growing or shrinking picture/image based on completion toward a predetermined goal (eg, UbiFit Garden)

Application: what operating system the mobile app can be accessed on for viewing the data/feedback

Software: the operating systems it can be accessed on if a piece of computing software is present for use at viewing the data

Website: can the data/feedback be viewed on a website?

Goal-setting capability: can predetermined goals be set by the user?

Customization: can the device or mobile app be customized by the end user (eg, via a software development kit)?

Each device was given a score between 1 and 6 for each attribute of behavior change. This score was used to describe two factors: (1) whether or not that device contained that behavior change attribute and (2) to what extent it did or did not contain the attribute. The self-monitoring scoring system that was used for each attribute was (1) yes; (2) yes, difficulties (eg, proximity to computer); (3) yes, lack of evidence to suggest this; (4) no, but present in future iterations; (5) no, but possible (with application programming interface or software development kit); and (6) not described/featured.

This scoring system was meant to be a descriptive tally of the behavior change attributes and not a judgment on the effectiveness of the various features.

## Results

### Review Statistics

**Figure 1:**
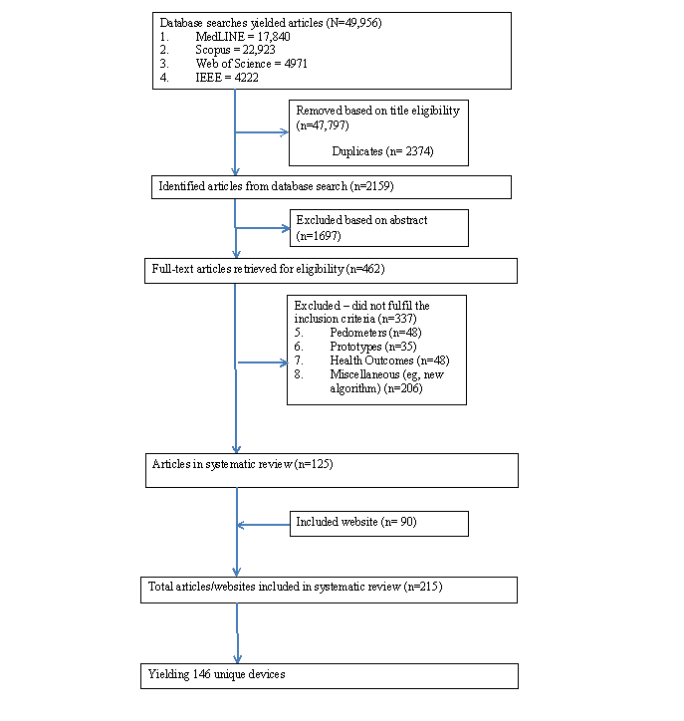
Study/website selection.

Database searches identified 49,956 articles (Figure 1), of which 462 were deemed to be potentially relevant and were retrieved for full-text analysis. Articles were excluded for a number of reasons (n=337):

1. Pedometer studies: these were excluded if no evidence could be found that the pedometer in question provided temporally stamped data.
2. Prototypes: if the device was not commercially available or if no data currently existed for the prototype and only proof of concept information was available were excluded.
3. Health outcome: articles were excluded if they examined the relationship between behavior (eg, sedentary behavior and/or PA) and a particular health outcome (eg, blood pressure, lipid profile) and the measurement tool of choice was not the main focus of the article.
4. Miscellaneous: articles were excluded if the purpose of the study was to examine a new algorithm or data processing procedure for device analysis.

The remaining 125 studies (on 46 devices) and 90 websites yielded 146 devices (see Multimedia Appendix 2) that were selected for detailed scrutiny. Of these, 64 were further removed because there was no evidence that they were designed for, had been used in, or could readily be modified for real-time self-monitoring purposes or that they were not currently available for purchase.

The remaining 82 [39-119] technologies were included in this review. Of these, 73 [39-110] technologies measured / self-monitored PA, of which 16 [43,45,55,56,58,62-66,81,86,90,91,94,103,107-109] provided some measure of physical inactivity (see Multimedia Appendix 3). In all, 9 [111-119] technologies measured self-monitored sedentary time (Multimedia Appendix 4), 8 [111,112,114-119] of which measured both PA and sedentary time.

### Physical Activity Self-Monitoring Technologies

**Figure 2:**
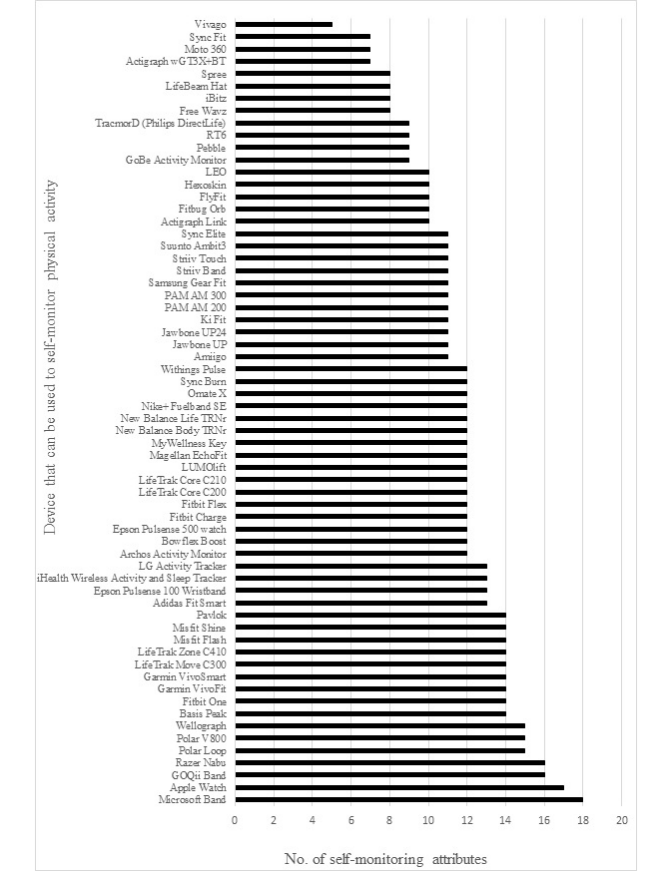
Technologies found that can be used to self-monitor and provide feedback on PA ordered by number of self-monitoring attributes that were found to be present in the technologies.

**Figure 3:**
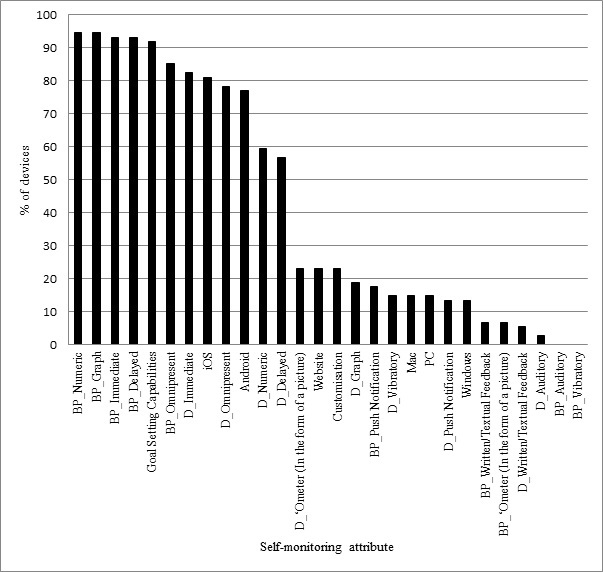
The proportion of devices that could be used to self-monitor and provided feedback on physical activity with specific self-monitoring attributes.

Figure 2 displays the number of self-monitoring attributes apparent in each of the devices found to measure/self-monitor PA. The device with the highest number of feedback attributes was the Microsoft Band [77] with 18 of 28 feedback possibilities that were coded. The most common feedback attribute used in the devices found was joint numeric and graphical data feedback on the associated backend platform, with 94% of the devices that self-monitored PA displaying these attributes. The least common form of feedback attribute was auditory feedback from the device (D_Auditory). This particular type of feedback was only present in 2% of cases (Figure 3).

### Sedentary Time Self-Monitoring Technologies

**Figure 4:**
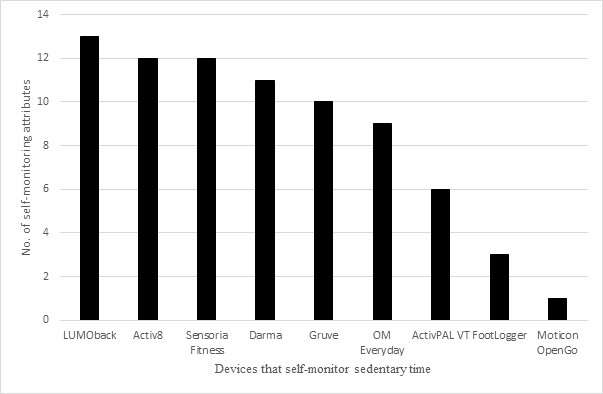
Technologies found that could be used to self-monitor and provided feedback on sedentary time ordered by number of feedback elements in the technologies.

**Figure 5:**
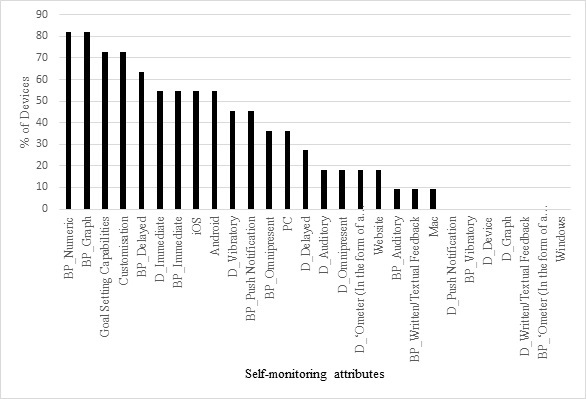
The proportion of devices that could be used to self-monitor and provided feedback on sedentary time with specific self-monitoring attributes.

Figure 4 displays the number of self-monitoring attributes apparent in each of the devices found to measure/self-monitor sedentary time. Figure 5 documents the popularity of the self-monitoring attributes with sedentary time self-monitoring devices. The device with the highest number of feedback attributes was the Lumo Back posture sensor and feedback coach [17] with 13 of 28 feedback possibilities that were coded. The most common feedback attribute used in the devices found was joint numeric and graphical data feedback on the associated backend platform, with 81% of the devices that self-monitor sedentary time displaying these attributes. The least common form of feedback attribute was push notification of feedback from the device of sedentary time on the device. This particular type of feedback was not present in any of the devices found.

## Discussion

The present systematic review sought to identify current measurement technologies available that could be used for real-time self-monitoring of sedentary time and/or PA. The review identified 125 articles on 46 device and 90 websites, for a combined total of 146 technologies that monitor sedentary time and/or PA. Of these, 82 devices were considered capable of self-monitoring sedentary time and/or PA. These devices can be used by researchers, clinicians, and the general public.

Technologies that self-monitor PA mainly come from the consumer health and fitness market. In general, these devices consist of an accelerometer for activity measurement (steps, calories burned, distance traveled) with varying secondary sensors, including gyroscope, inclinometer, lux sensors, skin sweat sensors, and other sensors that provide additional pieces of information. However, these devices will provide feedback only on PA and increases in PA do not automatically lead to decreases in sedentary time [120]. Additionally, more and more of these devices are providing feedback on not only the amount of PA, but also the length of time spent inactive.

There are devices from both the commercial and research sectors that self-monitor sedentary behavior. These devices tend to measure sedentary time in two different ways. Firstly, posture sensors measure sedentary time either through an accelerometer in conjunction with gravitational components and proprietary algorithms (eg, activPAL) or through the alignment of the area of the body surrounding the pelvic area (ie, pelvic alignment is different depending on standing, sitting, and lying). The other way technologies tend to measure sedentary time is via pressure sensors. These pressure sensors are either located in a sock, shoe, or chair. When placed in a sock or shoe, the pressure can determine standing when there is pressure on the sensor and when there is less pressure the wearer is sitting or lying. Located on a chair, there is a simple binary outcome: when the pressure sensor is active the user is sitting and when it is inactive there is no sitting behavior at that site.

Both these types of devices usually provide feedback either via vibratory feedback (eg, Jawbone UP) or via an omnipresent display on the device (eg, Garmin Vivofit). These devices tend to, but not exclusively, connect to a mobile app for feedback on the PA and sedentary time. For PA, this usually takes the form of energy expenditure or proprietary company points (eg, Nike Fuel). For sedentary time, this usually takes the form of time spent sitting (eg, LumoBack) These mobile apps allow the wearer to receive real-time continuous feedback along with goal-setting capabilities and customization of type and timing of feedback; this is an aspect not traditionally offered by research devices.

**Figure 6:**
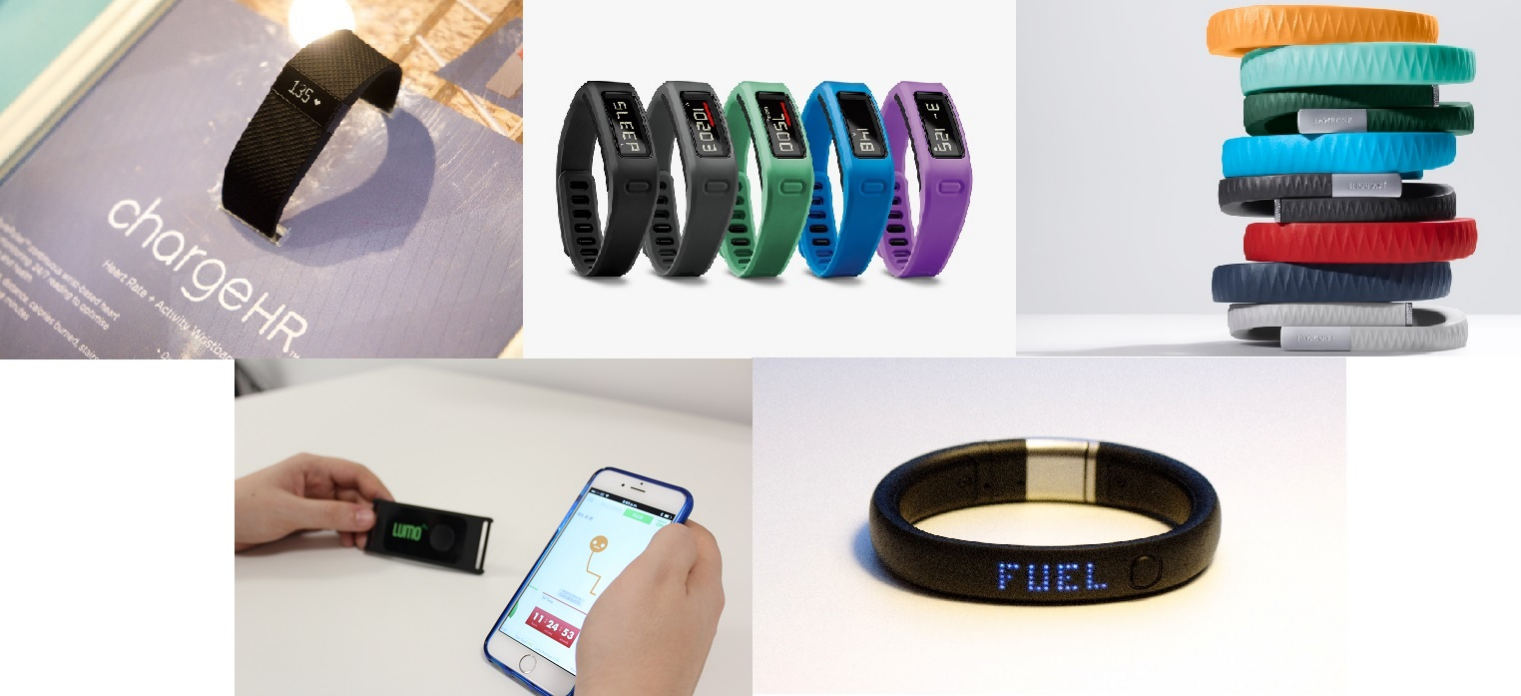
Example of devices discussed in this review. Clockwise from top left: Fitbit Charge HR, Garmin Vivofit, Jawbone Up, Nike Fuelband SE, Lumo Back Posture Sensor, and a mobile app.

With the plethora of devices now available (see Figure 6 for an example of popular devices), with differing attributes and cost, it is unsurprising that these devices are growing in popularity. However, and perhaps paradoxically, there are a small number of devices specifically designed to measure sitting time. Furthermore, the small number of devices that do provide feedback on sitting were not either originally designed for its measurement (eg, LumoBack) or are still primarily research tools to be used in scientific study (eg, ActivPAL VT).

Self-monitoring technologies need to provide real-time feedback on aspects of PA and sitting that are personalized and relevant to the individual (ie, the attributes of real-time feedback must resonate with the individual and not be simply information that has been presupposed for them). Additionally, the immediate feedback should be of a low cognitive load so that it can resonate immediately with the end user [121,122]. For example, the Fitbit one has a growing flower as a feedback indication of progression toward a user-defined goal. Using a pictorial representation of this nature will resonate easier with the user [123,124]. However, more detailed information on the temporal patterning of the behavior, for example, should be accessible from a mobile app, website, or software. Furthermore, the likelihood of the feedback being acted upon could be increased if it is provided in a manner that is context aware. In other words, the feedback must be given at a time when it can be acted upon by the user. For example, to reduce sitting, it should provide feedback while watching television rather than sitting in an exam or during a prolonged dental procedure. If these attributes could be integrated into a single device, it would help facilitate its use by differing populations regardless of technological ability. These devices need to have a substantial battery life and memory capacity at a reasonable cost. For this to occur, there is a need for cooperative work across different research disciplines and commercial fields to develop these context-aware, personalized feedback devices.

Not every user will have the same needs and the presentation of actionable information will need to be tailored to fit individual needs. In addition, simply providing more medical data to patients not only fails to guarantee improved outcomes, but also could potentially lead to negative consequences [125]. Activity trackers have poor evidence of prolonged use, with a conservatively estimated one-third discontinuing use by 6 months after initiation [126]. A recent study of several tools to encourage medication adherence in older adults, a major area of focus of mHealth developers, found that the most common descriptors participants used to describe their experience with the devices were “frustrating” and “challenging” [127]. In another study of the usage of a dietary app to promote healthy eating, investigators found that fewer than 3% used the app for at least 1 week and fewer than 10% of these individuals made positive changes in their diet [128]. Users require consumer-friendly devices and apps that are self-reinforcing and enjoyable to use. These goals might be accomplished with the use of incentives, gamification, and social networks to promote managed competition/cooperation among peers or family members.

In order for the promise of wearable technology to be fully realized, consumers, providers, and health care systems must be able to trust the reliability, privacy, and security of their data as well as the devices that collect and share it. Although regulatory oversight is often considered to be an impediment to the rapid propagation of innovative technologies, the existence of potential scams that could harm the end user dictates the need for some level of oversight. Globally, there is a great deal of uncertainty around wearable technology regulation; there are numerous countries that have no regulatory framework, whereas the other countries that do have a framework are still in their infancy and being actively refined [129,130].

Wearable technology users are also concerned about the privacy and ownership of their health data. In the era of big data, it is critical that the terms of ownership of personal data, most especially medical data, be unmistakably stated—not buried in the commonly unread and then accepted terms of use agreements—with users required to explicitly consent whenever their data are sold or transmitted to others [131].

One of the benefits of mHealth is easier accessibility to pertinent health care data, but this increased availability to both consumers and providers creates the potential for substantial security risks. Because of the small size of the device, it becomes easier to inadvertently lose or easier to steal, which may mean that the information stored on the device becomes accessible to others.

As consumer demand for wearable sensor increases, health care providers will face the possibility of being inundated by a flood of patient data. This will create a number of difficult challenges, including the potential requirement for 24/7 oversight, the need to summarize multiparameter, continuously collected data into a usable and clinically meaningful format [132].

The strengths of this review are the systematic approach taken and the comprehensive range of technologies found. However, there are some limitations. Due to the nature of articles included, it was not possible to present data on the validity and reliability of the devices in their ability to measure sedentary time. Similarly, due to the fact that self-monitoring using objective measurement tools is in its infancy, there are gaps in the literature as to whether these devices truly work as self-monitors; consequently, we cannot comment on how useful or valid they are in these settings. However, validity data are important. Users of self-monitoring technologies must be able to trust in the feedback that is being returned to them otherwise they may become disenfranchised with the tool and the behavior change tool. Therefore, incorporating important valid data with the feedback tools means additional value can be added to the consumers and potentially more potent behavior change.

In conclusion, the authors believe that this review is the first of its kind to systematically describe the wide breadth of devices that self-monitor and provide feedback on PA and sedentary behavior. There has been an explosion in the number of devices that measure PA and there is a greater need for the development of tools that specifically measure sitting time. Cooperative work between engineers, computer scientists, and academics in relevant fields is needed to develop these technologies that provide real-time, personalized, context-aware feedback to aid in the reduction in sitting time and its detrimental effect on cardiometabolic health independent of PA. This could potentially lead to the use of these devices in a health care setting as part of the increasing value-based care systems that are starting to arise in the United States or as a diagnostic tool, which is beginning to be implemented in the National Health Service in the United Kingdom.

This scoping review provides a record of a plethora of devices with information on their capabilities both in terms of their ability to measure behavior and to provide feedback to the user, providing a foundation for clinical, research, and public health use. Future studies are needed to further investigate the validity of these devices and their feasibility in increasing PA and/or decreasing sedentary time and the public health impact this may produce.

## Appendix

Multimedia Appendix 1 Search Strategy.

Multimedia Appendix 2 Supplementary Table 1 - All Devices.

Multimedia Appendix 3 Supplementary Table 2 - Devices that self monitor physical activity.

Multimedia Appendix 4 Supplementary Table 3 - Devices that Self-monitor Sedentary behaviour.

## Abbreviations

PA: physical activity
MVPA: moderate-to-vigorous physical activity
