# Supplementary material for: Devices for Self-Monitoring Sedentary Time or Physical Activity: A Scoping Review
Source: J Med Internet Res. 2016 May 4;18(5):e90. doi: 10.2196/jmir.5373 (PMC4871995; doi:10.2196/jmir.5373)
Supplement: Multimedia Appendix 1 [file jmir_v18i5e90_app1.pdf]

Appendix 1 - Search Strategy example – MEDLINE (search result in brackets)

Behaviour

1. MH "sedentary lifestyle" (1,560)
2. AB "Sedent* behav*" OR TI "sedent* behav*" (1,390)
3. AB Sedent* OR TI Sedent* (15,246)
4. AB "TV View*" OR TI "TV view*" (432)
5. AB "Video games" OR TI "Video games" (683)
6. AB "screen time" OR TI "screen time" (344)
7. AB "sedentary lifestyle" OR TI "sedentary lifestyle" (1,531)
8. AB "computer use" OR TI "computer use" (953)
9. AB "couch potato" OR TI "couch potato" (24)
10. AB "light activit*" OR TI "light activit*" (186)
11. AB "physical activ*" OR TI "physical activ*" (48,221)
12. AB "physical inactiv*" OR TI "physical inactiv*" (3,371)
13. AB "physical fit*" OR TI "physical fit*" (5,022)
14. MH Exercise OR TI exercise OR AB exercise (180,875)
15. AB "moderate-vigorous physical activ*" OR AB "moderate£vigorous physical activ*" OR AB "moderate#vigorous physical activ*" (67)
16. TI "moderate-vigorous physical activ*" OR TI "moderate£vigorous physical activ*" OR TI "moderate#vigorous physical activ*" (5)
17. TI MVPA OR AB MVPA (728)
18. TI "energy expenditure" OR AB "energy expenditure" (15,039)
19. MH "motor activity" OR AB "motor activity" OR TI "motor activity" (51,841)
20. MH "activities of daily living" OR AB "activities of daily living" OR TI "activities of daily living" (51,841)

21. AB Posture OR TI Posture (18,409)
22. S1 OR S2 OR S3 OR S4 OR S5 OR S6 OR S7 OR S8 OR S9 OR S10 OR S11 OR S12 OR S13 OR S14 OR S15 OR S16 OR S17 OR S18 OR S19 OR S20 (356,020)

Measurement

1. AB Validation OR TI Validation OR MH Validation (84,533)
2. AB Reliability OR TI Reliability (87,609)
3. AB "activ* monitor*" OR TI "activ* monitor*" (2,213)
4. AB "objective measur*" OR TI "objective measur*"(8,708)
5. AB "device*" OR TI "device*" (201,464)
6. AB Sensor OR TI Sensor OR MH Sensor (40,263)
7. AB "wear* monitor*" OR TI "wear* monitor*" (45)
8. AB Methodolog* OR TI Methodolog* (161,958)
9. AB Assessment OR TI Assessment (499,305)
10. AB "Motion Sensor*" OR TI "Motion Sensor*" (334)
11. AB "Physiological Sensor*" OR TI "Physiological Sensor*" (89)
12. AB "Ambulatory monitor*" OR TI "Ambulatory monitor*" (1,933)
13. S22 OR S23 OR S24 OR S25 OR S26 OR S27 OR S28 OR S29 OR S30 OR S31 OR S32 OR S33 (1,001,341)

Together S35 AND S36 (41,991)

Limiters – English Language, Human, All Adult 19+ years

Total Number = (17,840)
